# Integrating a Soft Body Diode in the Super-Junction MOSFET by Using an n^−^/n^+^-Buffer Layer

**DOI:** 10.3390/mi13122193

**Published:** 2022-12-10

**Authors:** Zhi Lin, Wei Zeng, Da Wang, Ping Li, Shengdong Hu

**Affiliations:** School of Microelectronics and Communication Engineering, Chongqing University, Chongqing 400044, China

**Keywords:** super-junction MOSFET, buffer layer, body diode, reverse recovery, oscillation

## Abstract

In this paper, a novel silicon super-junction (SJ) MOSFET embedded with a soft reverse recovery body diode is proposed and studied by numerical simulation. The device introduces an n^+^-buffer layer between the n^−^-buffer layer and the n^+^-substrate to improve the reverse recovery behaviour of its body diode. The n^+^-buffer layer provides residual carriers during the reverse recovery process, reduces the overshoot voltage, and suppresses oscillation. Simulated results demonstrate that the increment of the on-resistance and the drain-to-source overshoot voltage can be respectively kept below 5% and 20 V, if a 10 μm n^+^-buffer layer whose impurity concentration ranges from 4 × 10^15^ cm^−3^ to 6 × 10^16^ cm^−3^ is used. In addition, the fabrication process is the same as that of the conventional SJ-MOSFET. These features make the proposed SJ-MOSFET suitable for inverter applications.

## 1. Introduction

The performance of the traditional silicon-based power MOSFET is limited by the contradictory relationship between its specific on-resistance and breakdown voltage, which is known as the silicon limit [1]. In order to overcome this limitation, new semiconductor materials are adopted and novel device structures are developed. Power MOSFETs made from the third-generation semiconductors, such as the SiC MOSEFT and GaN HEMT, have demonstrated their superior performance over their silicon-based counterparts [2,3]. On the other hand, the super-junction (SJ) MOSFET breaks the silicon limit by using alternative highly doped n-pillars and p-pillars in its drift region [1,4,5,6]. The most attractive advantage is that its specific on-resistance is less than one fifth of that of the conventional power MOSFET with the same rated voltage. As a result, the device area and parasitic capacitors are reduced. These features make it suitable for high-frequency circuits. Therefore, it is now widely used in modem power converters [7]. One of its disadvantages is the poor reverse recovery behaviour of its body diode, which can be used as the free-wheeling diode in power inverters [8]. In inverter circuits with an inductance load, free-wheeling diodes are necessary to conduct load currents when power devices are switched off. There are two main problems with its body diodes: the reverse recovery charge is large, and the reverse recovery current is snappy [9]. The former causes high energy loss and reduces power efficiency. The latter causes overshoot voltage and oscillation. The reverse recovery problem of the body diode is a key scientific problem toward super-junction MOSFETs. It limits their applications in power inverters. Many solutions have been proposed to reduce the reverse recovery charge, such as lifetime killing techniques using irradiation by electrons, protons, helium ion and heavy metal doping by gold, platinum [10,11]. Some researchers suggest integrated unipolar diodes [12,13]. Little attention has been paid to the snappy reverse recovery current. Modern SJ-MOSFETs usually use a lightly doped n^−^-buffer layer to improve the softness of the reverse recovery current [14]. The thicker the n^−^-buffer layer, the softer the reverse recovery current [15]. However, a thick n^−^-buffer layer increases the on-resistance of the SJ-MOSFET.

In this study, we propose a practical solution to integrate a soft body diode in the SJ-MOSFET without compromising its on-resistance. It uses an additional n^+^-buffer layer to reduce the overshoot voltage and suppress oscillation during the reverse recovery process of the body diode. The device structure and mechanism are shown in Section 2. Section 3 summarizes the optimised results. The conclusion is in Section 4.

## 2. Device Structure and Mechanism

**Figure 1:**
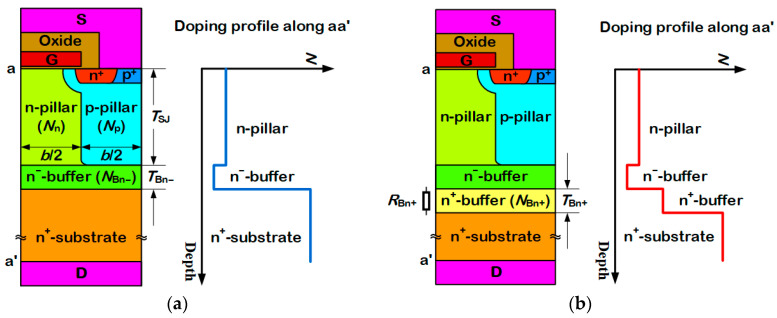
Schematic cross-sections and doping profiles along the n-pillar center of (**a**) the conventional SJ-MOSFET and (**b**) the proposed SJ-MOSFET. An n^+^-buffer layer is introduced in the proposed SJ-MOSFET. Its impurity concentration is much lower than that of the n^+^-substrate but much higher than that of the n^−^-buffer layer.

Figure 1 shows the schematic cross-sections of the conventional SJ-MOSFET and the proposed SJ-MOSFET, which both have a symmetrical interdigitated layout. The difference lies in the buffer region. As shown in Figure 1b, an n^+^-buffer layer is added between the n^−^-buffer layer and the n^+^-substrate in the proposed structure. Its impurity concentration is much lower than that of the n^+^-substrate but much higher than that of the n^−^-buffer layer. It is used to improve the softness of the body diode. As explained before [9], the reverse recovery current of the conventional SJ-MOSEFT is snappy because there are not enough carriers when it reaches its negative peak value. Since the n^+^-substrate is heavily doped and the net recombination rate in the semiconductor is proportional to the carrier density [16], the holes’ density in the n^+^-substrate is tiny. Once the pillars are depleted, only a few carriers exist in the n^−^-buffer layer. Then, the reverse recovery current drops sharply. In the proposed SJ-MOSEFT, since the n^+^-buffer layer has a much lower impurity concentration than the n^+^-substrate, some nonequilibrium carriers accumulate in this region when the body diode is forward biased. Then, during the reverse recovery process, these carriers keep the current continuous after the pillars are depleted. On the other hand, the n^+^-buffer layer introduces the series resistance. To reduce its impact on the on-resistance and the breakdown voltage, its impurity concentration should be higher than those of pillars and the n^−^-buffer layer.

In the following simulations, 650 V-rated silicon SJ-MOSFEFTs with a half cell pitch of *b* = 6 μm are used. The pillar thickness *T*_SJ_ is 40 μm. For simplicity, SJ pillars are uniformly doped. The p-pillar concentration *N*_p_ and the n-pillar concentration *N*_n_ are both set to 4 × 10^15^ cm^−3^. The thickness of the n^−^-buffer layer *T*_Bn-_ is 10 μm. Its impurity concentration *T*_Bn-_ is 1 × 10^15^ cm^−3^. The thickness and the impurity concentration of the n^+^-buffer layer, namely *T*_Bn+_ and *N*_Bn+_, are respectively chosen as 10 μm and 4 × 10^16^ cm^−3^, unless otherwise specified. Each device has an area of 10 mm^2^. The Sentaurus Device simulator [17] is used to analyse the electrical performances of devices. Employed mobility models include the DopingDenpendnce model, the HighFieldSturation model, and the Enormal model. The Shockley–Read–Hall recombination model and the Auger recombination model are also used. The Okuto avalanche model is used in blocking characteristics’ analysis. The electron lifetime and the hole lifetime are, respectively, 3 μs and 1 μs.

**Figure 2:**
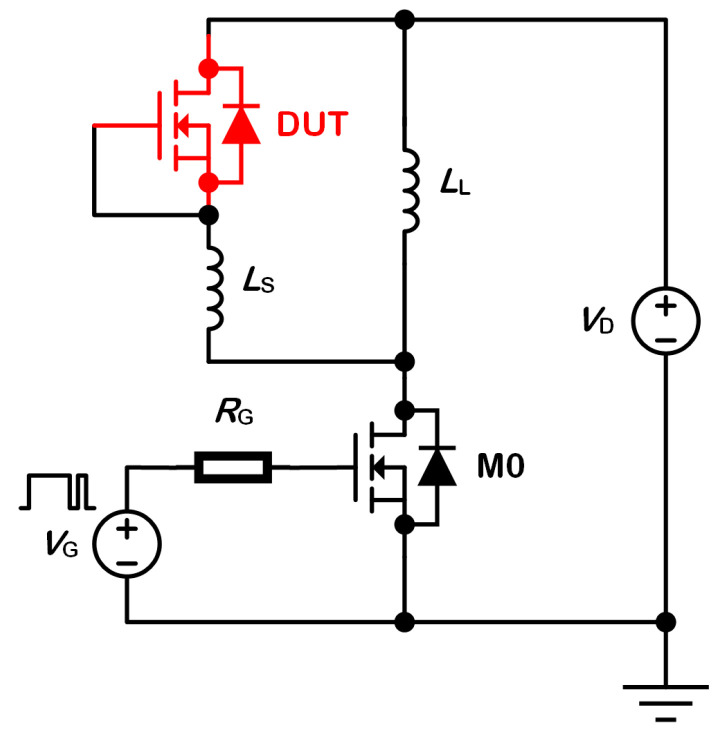
Schematic circuit configuration of the double pulse test.

To explore the reverse recovery behaviour of body diodes, the double pulse test is carried out in mix-mode simulations. It uses the circuit shown in Figure 2. The Device Under Test (DUT) and the switching transistor M0 are both constructed with finite element models. The DUT is marked in red. To close the channel, its gate electrode and source electrode are connected. Then, its body diode serves as the free-wheeling diode. Other components are constructed with compact models. *L*_D_ and *L*_S_ are the load inductor and the parasitic inductor, respectively. *R*_G_ is the gate resistor. It controls the commutation velocity *di*/*dt* during the reverse recovery process. *V*_D_ is bus voltage source. *V*_G_ drives M0 with a double pulse signal.

## 3. Results and Discussion

**Figure 3:**
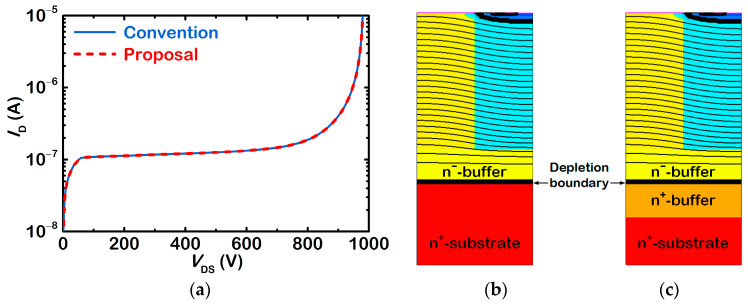
(**a**) Compared blocking characteristics of the two SJ-MOSFETs. Equipotential lines in (**b**) the conventional SJ-MOSFET and (**c**) the proposed SJ-MOSFET at *V*_DS_ = 650 V.

Figure 3a compares the simulated blocking characteristic of the proposed SJ-MOSFET with that of the conventional SJ-MOSFET. Figure 3b,c show respective equipotential lines at *V*_DS_ = 650 V. The extracted breakdown voltage of the conventional SJ-MOSFET is 979.5 V, while that of the proposed device is 979.6 V. Their blocking characteristics are almost the same, because their voltage-sustaining layers, namely the pillars and the n^−^-buffer layer, are the same. The n^+^-buffer layer is hardly contributed to the breakdown voltage due to its high impurity concentration. As shown in Figure 3c, the depletion region in the n^+^-buffer layer is negligible.

**Figure 4:**
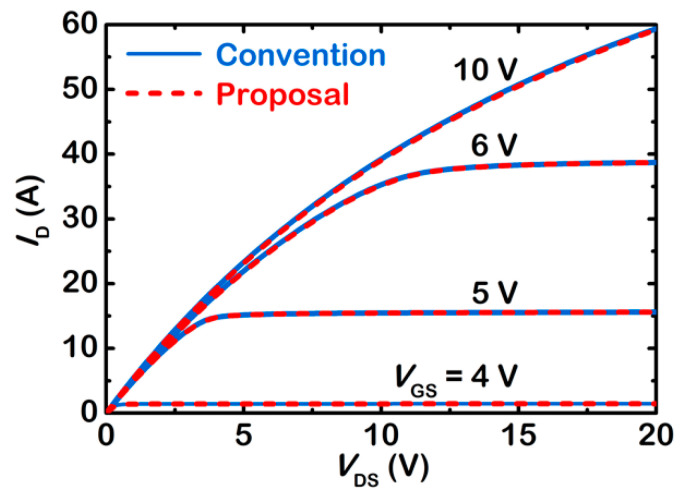
Compared output characteristics of the two SJ-MOSFETs.

Figure 4 compares output characteristics of the two SJ-MOSFETs. It is demonstrated that the on-state current of the proposed SJ-MOSFET is a little smaller than that of the conventional device. The on-resistance, *R*_ON_, of the proposed SJ-MOSFET and conventional SJ-MOSFET, extracted at *V*_GS_ = 10 V, are 207.7 mΩ and 209.3 mΩ, respectively. The increment of on-resistance, Δ*R*_ON_, is 1%. Δ*R*_ON_ is defined as the percentage increase in on-resistance with respect to the conventional device

(1) $$\Delta R_{\mathrm{ON}}=\frac{R_{\mathrm{ON},\;\mathrm{proposal}}-R_{\mathrm{ON},\;\mathrm{convention}}}{R_{\mathrm{ON},\;\mathrm{convention}}}\times 100\%$$

where *R*_ON, proposal_ and *R*_ON, convention_ are on-resistances of the proposed SJ-MOSEFT and the conventional SJ-MOSEFT, respectively. This increment comes from the series resistance of the n^+^-buffer layer *R*_Bn+_, as illustrated in Figure 1b. Since the impurity concentration of the n^+^-buffer layer is lower than that of the n^+^-substrate, its series resistance is also larger. *R*_Bn+_ can be simply calculated as

(2) $$R_{\mathrm{Bn}+}=\frac{1}{q\mu_{n}A}\frac{T_{\mathrm{Bn}+}}{N_{\mathrm{Bn}+}}$$

where *q* is electron charge, *μ*_n_ is the electron mobility, and *A* is the area of the device. Hence, *R*_Bn+_, as well as Δ*R*_ON_, increases with *T*_Bn+_ and decreases with *N*_Bn+_.

**Figure 5:**
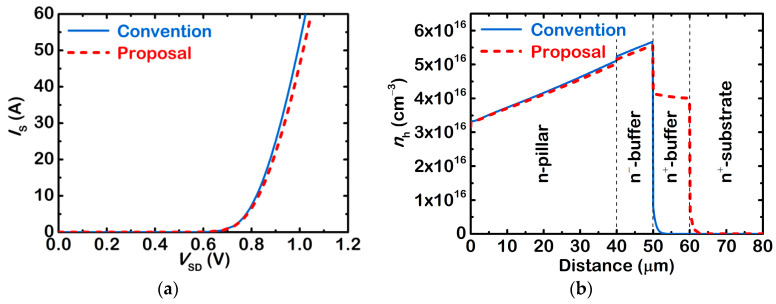
(**a**) Compared forward *I*-*V* curves of the two body diodes. (**b**) Hole density distributions along the n-pillar centre at *I*_S_ = 20 A.

Figure 5a shows the simulated forward *I*-*V* curves of the two body diodes. At *I*_S_ = 20 A, the extracted voltage drops are 0.82 V and 0.83 V, respectively. Again, the n^+^-buffer layer introduces the series resistance and increases the voltage drop. The corresponding hole density distributions along the n-pillar centre are shown in Figure 5b. For the conventional device, since both the SJ pillars and the n^−^-buffer layer are lightly doped, a large amount of non-equilibrium carriers accumulated. The injected holes vanish quickly in the n^+^-substrate because the recombination rate is very high in this heavily doped region. For the proposed device, since the n^+^-buffer layer is not heavily doped, the recombination rate is much smaller than that in the n^+^-substrate. Thus, many non-equilibrium carriers also accumulate there. These additional carriers help to the improve the reverse recovery behaviour of its body diode.

**Figure 6:**
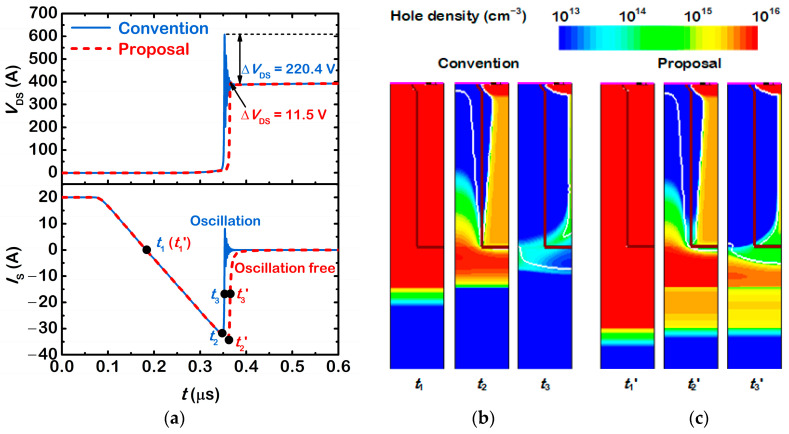
(**a**) Simulated reverse recovery waveforms of the two body diodes. Evolution of hole density distributions in (**b**) the conventional device and (**c**) the proposed device during the reverse recovery process.

Figure 6a shows reverse recovery waveforms with *di*/*dt* = 200 A/μs and *L*_S_ = 10 nH, at 300 K. *V*_D_ is 400 V. It is obvious that the reverse recovery current of the proposed device is softer than that of the conventional device. During the reverse recovery period, the maximum commutation velocity *di*/*dt*_max_ decreases by 62.9%, from 2.4 × 10^10^ A/s to 8.9 × 10^9^ A/s. As a result, the drain-to-source overshoot voltage Δ*V*_DS_ decreases from 220.4 V to 11.5 V. Figure 6b,c show hole density distributions at select points. Once the space charge region reaches the n^−^-buffer region at *t*_2_(*t*_2_′), the reverse recovery current reaches its negative peak value. After that, the reverse recovery current decreases gradually. For the conventional device, only a few holes exit in the SJ pillars and the n^−^-buffer layer. Then, its reverse recovery current decays steeply, which causes a large voltage spike. On the other hand, for the proposed device, there are still plenty of holes in the n^−^/n^+^-buffer layer. Therefore, the reverse recovery current decays slowly and the voltage spike is suppressed. Of course, it takes some time to extract these residual holes. Thus, the reverse recovery charges increase. The reverse recovery behaviour of the proposed device can be improved further by increasing the thickness, or decreasing the impurity concentration of the n^+^-buffer layer.

Table 1 summarizes performances of the above two SJ-MOSFETs. The transfer characteristics of the two SJ-MOSEFT are not exhibited. Their threshold voltages are equal because they have the same MOS channel structure. The most affected parameters are *R*_ON_ and Δ*V*_DS_ (or *di*/*dt*_max_). They are contradictory. Increasing *T*_Bn+_ or decreasing *N*_Bn+_ introduces more non-equilibrium carriers in the n^+^-buffer layer. Then, both *di*/*dt*_max_ and Δ*V*_DS_ are reduced. However, *R*_ON_ also increases, as Equation (2) demonstrates. For example, Δ*V*_DS_ can be reduced to 9.9 V by increasing *T*_Bn-_ from 10 μm to 15 μm, as listed in Table 1. However, *R*_ON_ increases to 229.4 mΩ. The increment exceeds 10%. Table 1 also includes a SJ-MOSEFT with reduced *Q*_RR_; but, its *V*_F_ and Δ*V*_DS_ increases. In the rest of this section, we discuss the influence of *N*_Bn+_ and *T*_Bn+_ on Δ*V*_DS_ and Δ*R*_ON_.

**Figure 7:**
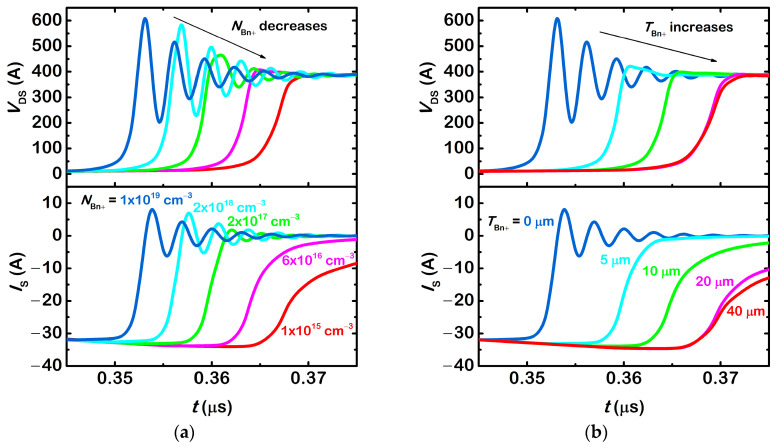
(**a**) Reverse recovery waveforms of body diodes with various *N*_Bn+_. *T*_Bn+_ = 10 μm for all devices. (**b**) Reverse recovery waveforms of body diodes with various *T*_Bn+_. *N*_Bn+_ = 4 × 10^16^ cm^−3^ for all devices.

Figure 7a shows reverse recovery waveforms of devices with *T*_Bn+_ = 10 μm and *N*_Bn+_ ranges from 1 × 10^19^ cm^−3^ to 1 × 10^15^ cm^−3^. *N*_Bn+_ = 1 × 10^19^ cm^−3^ represents the conventional SJ-MOSFET. As *N*_Bn+_ decreases, the reverse recovery current becomes smoother. Both *di*/*dt*_max_ and Δ*V*_DS_ decreases. Oscillation is also gradually suppressed. This is because the net recombination rate in the semiconductor is proportional to the carrier density. If *N*_Bn+_ decreases, the net recombination rate also decreases. Then, more holes accumulate in the n^+^-buffer layer when the body diode is forward biased. During the reverse recovery process, they make the reverse recovery current smoother. Figure 7b shows reverse recovery waveforms of devices with *N*_Bn+_ = 4 × 10^16^ cm^−3^ and *T*_Bn+_ ranges from 0 μm to 40 μm. *T*_Bn+_ = 0 μm represents the conventional SJ-MOSFET. As *T*_Bn+_ increases, the reverse recovery current becomes smoother. Both *di*/*dt*_max_ and Δ*V*_DS_ decreases. Oscillation is also gradually suppressed. This is because more holes accumulate in the n^+^ buffer layer when the body diode is forward biased if *T*_Bn+_ increases. During the reverse recovery process, they make the reverse recovery current smoother. The improvement becomes insignificant once *T*_Bn+_ exceeds 20 μm. The reason is that holes deep into the n^+^ buffer layer recombine with electrons during the reverse recovery process. Their contribution to the reverse recovery current is negligible.

**Figure 8:**
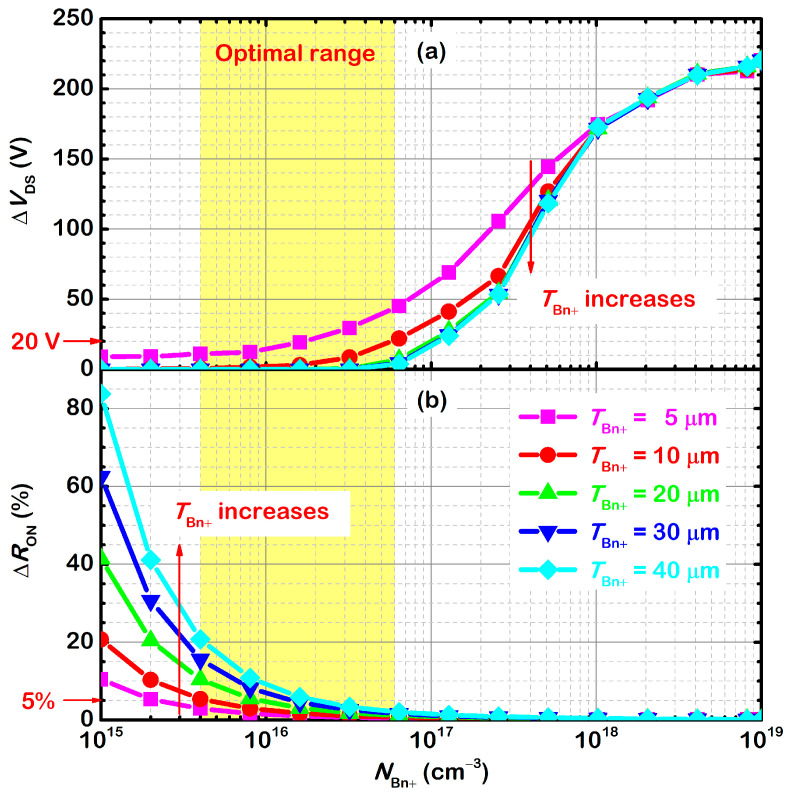
Influence of *N*_Bn+_ and *T*_Bn+_ on (**a**) Δ*V*_DS_ and (**b**) Δ*R*_ON_.

Figure 8 summarizes the influence of *N*_Bn+_ and *T*_Bn+_ on Δ*V*_DS_ and Δ*R*_ON_. *T*_Bn+_ increases from 5 μm to 40 μm. For each *T*_Bn+_, *N*_Bn+_ ranges from 1 × 10^15^ cm^−3^ to 1 × 10^19^ cm^−3^. If *N*_Bn+_ = 1 × 10^19^ cm^−3^, the n^+^-buffer layer merges with the n^+^-substrate. Then, all samples are the same with the conventional SJ-MOSFET. If *N*_Bn+_ = 1 × 10^15^ cm^−3^, the n^+^-buffer layer merges with the n^−^-buffer layer. Increasing *T*_Bn+_ is equivalent to increasing the thickness of the n^−^-buffer layer. This is the usual method used in conventional SJ-MOSFETs. Without a doubt, Δ*R*_ON_ increases rapidly. For the proposed SJ-MOSFET with the same *T*_Bn+_, Δ*V*_DS_ increases, and Δ*R*_ON_ decreases with *N*_Bn+_. When *N*_Bn+_ is below 2 × 10^16^ cm^−3^, Δ*V*_DS_ is ignorable but Δ*R*_ON_ is remarkable. The overshoot voltage is almost eliminated. When *N*_Bn+_ is above 1 × 10^17^ cm^−3^, Δ*R*_ON_ is ignorable but Δ*V*_DS_ is remarkable. For the same *N*_Bn+_, Δ*V*_DS_ decreases, and Δ*R*_ON_ increases with *T*_Bn+_. Figure 8 also shows that *N*_Bn+_ plays a more effective role in reducing Δ*V*_DS_ than *T*_Bn+_, especially when *T*_Bn+_ exceeds 10 μm. It means that a thick n^+^-buffer layer is not necessary. For *T*_Bn+_ = 10 μm, the optimal range of *N*_Bn+_ is 4 × 10^15^ cm^−3^ to 6 × 10^16^ cm^−3^. In this range, Δ*R*_ON_ is within 5% and Δ*V*_DS_ is within 20 V. Even *T*_Bn+_ increases to 40 μm, Δ*R*_ON_ is within 20% and Δ*V*_DS_ is within 4 V.

## 4. Conclusions

This study demonstrates that a soft reverse recovery body diode can be integrated into the SJ-MOSFET by adding an n^+^-buffer layer between the n^−^-buffer layer and the n^+^-substrate. During the reverse recovery process, the drain-to-source overshoot voltage Δ*V*_DS_ can be greatly reduced and oscillation can be eliminated. Compared with the conventional device that uses a thick n^−^-buffer layer, the proposal solution does not compromise the on-resistance too much. According to the simulated results, the increment of *R*_ON_ and the overshoot voltage can be, respectively, kept below 5% and 20 V, if a 10 μm n^+^-buffer layer whose impurity concentration ranges from 4 × 10^15^ cm^−3^ to 6 × 10^16^ cm^−3^ is used. Moreover, the fabrication process is the same as that of the conventional SJ-MOSFET.

## Figures and Tables

**Table 1:** Performance comparison of SJ-MOSFETs.

Device		BV (V)	*R*_ON_ (mΩ)	*V*_F_ (V)	*di*/*dt*_max_ (A/s)	Δ*V*_DS_ (V)	*Q*_RR_ (μC)
Convention	*T*_Bn−_ = 10 μm	979.5	207.7	0.82	2.4 × 10^10^	220.4	2.8
	*T*_Bn−_ = 15 μm	993.3	229.4	0.82	9.1 × 10^9^	9.9	3.2
[13]		720.0	178.8	0.95	-	141.2	0.3
Proposal		979.6	209.3	0.83	8.9 × 10^9^	11.5	3.4
